# Lowered Maternal and Paternal Plasma Concentrations of Choline Are Associated with the Severity of Congenital Heart Defects in the Offspring

**DOI:** 10.3390/nu18091455

**Published:** 2026-05-01

**Authors:** Rima Obeid, Annabelle Wagner, Celina Löhfelm, Jürgen Geisel, Hashim Abdul-Khaliq

**Affiliations:** 1 Department of Clinical Chemistry and Laboratory Medicine, Saarland University Hospital, Kirrberg Street, Building 57, D-66421 Homburg, Germany; celina.loehfelm@uks.eu (C.L.); juergen.geisel@uks.eu (J.G.); 2 Department of Pediatric Hematology and Oncology, Saarland University Hospital, Kirrberg Street, Building 9, D-66421 Homburg, Germany; annabelle.wagner@uks.eu; 3 Department of Pediatric Cardiology, Saarland University and University Hospital, Building 9, D-66424 Homburg, Germany; hashim.abdul-khaliq@uks.eu

**Keywords:** betaine, children, choline, congenital heart defects, folate

## Abstract

Background/Objectives: Congenital heart defects (CHDs) are associated with disruptions in one-carbon metabolism. In a family-based trio design, we investigated whether plasma concentrations of choline, betaine, and folate are associated with CHD severity. Methods: The study included 72 children with CHD, 69 of their mothers and 64 of the fathers. CHD clinical severity was classified according to the European network of population-based registries for the epidemiological surveillance of congenital anomalies (EUROCAT) system and the German PAN study (Prevalence of Congenital Heart Defects in Newborns). Concentrations of choline, betaine, and folates were quantified in plasma and urine samples from a subgroup of the participants. Results: The children [mean (SD) age 3.1 (3.2) years, 59.7% males] presented with varying CHD severities according to EUROCAT (62.5% severe and 37.5% mild) and PAN classifications (45.8% severe, 30.6% moderate and 23.6% mild). The means (SD) of plasma concentrations of choline were 14.0 (10.0) µmol/L in the children, 9.5 (5.1) µmol/L in the mothers and 10.3 (5.4) µmol/L in the fathers. Plasma choline concentrations < 10 µmol/L were observed in 38 mothers (66.7%) and were associated with having a child with severe CHD [adjusted odds ratio (aOR) 3.7; 95% confidence intervals (95%CIs) = 1.1, 12.2] compared to mothers with choline ≥ 10 µmol/L. Lowered plasma choline concentrations were detected in 27 fathers (62.8%) and were also associated with severe CHD (aOR 7.4; 95%CIs = 1.7, 31.5). Child concentrations of choline, betaine and folate and parents’ concentrations of betaine and folate were not associated with disease severity. Conclusions: Lower plasma choline in the parents detectable several years after conception was related to having a child with severe CHD compared to families of children with higher plasma choline. Maternal and paternal choline metabolism may have a role in modulating CHD severity. Etiological studies aiming at the prevention of congenital anomalies should focus on maternal and paternal risk factors in the preconception and early pregnancy.

## 1. Introduction

Congenital heart defects (CHDs) occur in approximately 1 in 100 live births and represent structural abnormalities of the heart walls, valves, or major blood vessels [1,2]. Although many defects can be treated with surgical or medical procedures, even mild to moderate forms of CHD are associated with increased morbidity and mortality [2]. Maternal risk factors for CHDs are poorly understood, while paternal risk factors are not well investigated.

Pregnancy, spermatogenesis and fetal development are characterized by markedly increased demands for folate and other nutrients involved in one-carbon (C1) metabolism. Observational studies have shown that the risk of having a child with CHDs was lower in women who used folic acid-containing supplements during pregnancy compared to non-supplemented women [3,4,5,6,7,8]. In addition, substantial evidence indicates that paternal risk factors can affect pregnancy outcomes. The father contributes not only 50% of the DNA of the fetus, but also molecular imprints such as genomic methylation patterns [9]. Massive genome DNA-hypomethylation and histone modifications take place during spermatogenesis which affect genomic imprinting [10], suggesting paternal-transmitted effects on birth outcomes. For example, parental folate status has been shown to affect fetal development in a rat model [11].

Folate provides purine and pyrimidine nucleotides and methyl groups for DNA synthesis and methylation reactions [12,13]. The choline/betaine pathway also contributes to cellular methylation, particularly under conditions of limited folate availability [14,15]. Choline is irreversibly oxidized to betaine (trimethylglycine), which donates methyl groups needed to convert homocysteine to methionine and dimethylglycine [15]. Homocysteine remethylation via betaine–homocysteine methyltransferases (BHMT and BHMT2) relies on methyl groups derived from choline/betaine (via BHMT) or folate pathways (via BHMT2 that utilizes S-adenosylmethionine) [16]. Choline has additional and distinct biological functions such as serving as a precursor for the neurotransmitter acetylcholine and as a structural component of membrane phospholipids.

Disturbances in choline metabolism have been linked to CHD in both clinical and experimental studies [17,18,19,20,21]. For instance, altered biosynthesis of phosphatidylcholine and phosphatidylethanolamine were detected in maternal urine samples collected between 14 and 37 weeks of gestation from women who later delivered children with CHD [19]. Furthermore, low first-trimester serum concentrations of phosphatidylcholine derivatives such as lysophosphatidylcholine and sphingomyelin may predict CHD in the offspring [22]. Mothers of children with CHD exhibit marked dysregulation of plasma choline-derived phospholipids not only during pregnancy, but also after birth [17,18,19]. Higher plasma concentrations of betaine and choline have also been reported in children affected with CHDs [mean (SD) age = 3.3 (0.2) years] compared with children of a similar age but without CHD [23]. Collectively, alterations in choline metabolism have been reported in women and their children at different developmental stages even outside the etiological time window of CHD (i.e., early pregnancy).

CHDs also exhibit high heritability estimates, ranging from 0.5 to 0.9 [24,25]. In line with this, maternal genetic variants affecting choline or folate metabolism have been linked to an increased risk of having a child with CHD [26,27,28]. Moreover, our previous study showed that both children with CHD [mean (SD) of age 15.9 (12.3) months] and their mothers had elevated concentrations of dimethylglycine, the demethylation product of betaine, compared with control children and their mothers, respectively [29]. Together, these observations support the hypothesis that familial metabolic programming may be transmitted from the parents to the child and contribute to CHD etiology.

The present study tested the following hypotheses: (1) low plasma concentrations of choline- or folate-related markers in the child are associated with CHD severity; and (2) low plasma concentrations of choline- or folate-related markers in the parents are associated with CHD severity in the child. Additionally, we aimed to examine if plasma folate concentrations in the children are associated with plasma choline and betaine and study determinates of urinary concentrations of choline metabolites in children with CHD.

## 2. Materials and Methods

### 2.1. Subjects

This study was conducted within the framework of the National Registers for Congenital Heart Defects, a nationwide genetic research initiative designed to collect biospecimens from children with CHD and their parents (trio samples). Parents and their children with CHD were recruited at the Department of Pediatric Cardiology, Saarland University Hospital, Germany, between January 2021 and May 2022. Consecutive children (age < 10 y) suffering from CHD of any severity were eligible for inclusion. Children with syndromic conditions were eligible. Parents were enrolled if they were present at the hospital and agreed to participate. Exclusion criteria were children conceived by in vitro fertilization, those affected with congenital metabolic disorders, and children after surgical procedures or acute illnesses. The children were recruited during a scheduled hospital admission prior to any invasive surgical or catheter-based intervention. A standardized questionnaire was completed by the parents and collected demographic data, obstetric history, smoking, alcohol consumption, medication use, and vitamin supplementation. Additional information was obtained on the timing of CHD diagnosis, birth weight, and pregnancy-related risk factors and complications. Information on parent’s dietary patterns (e.g., vegetarian) and frequency (per week) of consumption of meat, fish, dairy products, fruits and vegetables were also collected. CHD severity was classified according to the EUROCAT (European network of population-based registries for the epidemiological surveillance of congenital anomalies) system [30] and the German PAN study (Prevalence of Congenital Heart Defects in the Newborns or Prävalenz angeborener Herzfehler bei Neugeborenen) [1].

The study protocol was reviewed and approved by the ethic committee of Saarland (Ärztekammer des Saarlandes; approval number: 54/20, approval date 16 March 2020). The study was conducted in accordance with the ethical principles of the World Medical Association (Declaration of Helsinki) and the International Council for Harmonisation Good Clinical Practice (ICH-GCP) guidelines.

### 2.2. Blood Collection and Laboratory Analyses

Blood was collected from the children and their parents into EDTA-K^+^-containing tubes. Additionally, samples of spontaneous urine were collected from the children into dry tubes. The blood was centrifuged and the EDTA plasma was collected into clean Eppendorf tubes. All samples were stored at −80 °C until analyses of the biomarkers. The remaining EDTA blood samples were forwarded to the biobank of the Competence Network for Congenital Heart Defects in Berlin, Germany. Blood samples were missing from 17 children due to either technical difficulties during blood collection or a limited amount of blood and the need to prioritize the routine blood analyses. Blood samples were missing from 14 of the 64 fathers and 4 of the 69 mothers who participated in the study. The reasons for missing parents’ samples were unwillingness to provide a blood sample to the study or not attending the hospital visit.

Concentrations of plasma folate forms and plasma and urine choline and betaine were measured at the Department of Clinical Chemistry and Laboratory Medicine, Saarland University Hospital, Homburg, Germany. The concentrations of choline and betaine in plasma and urine were measured by using ultra performance liquid chromatography tandem mass spectrometry (UPLC-MS/MS) and isotope labelled d9-choline and d9-betaine chloride as internal standards as described elsewhere [31,32]. Urine samples were diluted 1:5 in water (20 µL urine and 80 µL water) before adding the acetonitrile–internal standards solution. EDTA plasma (100 µL) or diluted urine (100 µL) samples were mixed with 300 µL of acetonitrile containing the internal standards. After centrifugation at 10,000× *g* for 10 min at ambient temperature, the supernatant was collected into a clean vial and measured on the UPLC-MS/MS system. Samples from the children and the parents were measured in the same batch to minimize analytical variations. Acquity Ultra Performance LC system coupled with a MicroMass Quattro Premier XE tandem quadrupole mass spectrometer (Waters Corporation, Milford, MA, USA) was used for the measurements. Each sample preparation batch included three quality control samples to capture the day-to-day variations. The between-day coefficients of variation for the choline and betaine assay were <7%. The concentrations of creatinine in urine samples were measured by using the COBAS INTEGRA system (Roche Diagnostics, Mannheim, Germany). Concentrations of choline and betaine in urine were expressed as mmol/mol creatinine.

The individual folate forms were measured in EDTA plasma using a validated UPLC-MS/MS method as described elsewhere [31,33]. The method relies on isotope labelled folate forms and quantifies 5 different forms: (6S)-5-methyltetrahydrofolate (5-MTHF), (6S)-tetrahydrofolate, (6S)-5-formyltetrahydrofolate, folic acid, and (6R)-5,10-methelynetetrahydrofolate. Plasma total folate was calculated by summing of the individual forms. The none-methylfolate was the sum of all forms except 5-MTHF. The between-day CVs were 2% for 5-methyltetrahydrofolate and up to 11.2% for the minor folate forms.

### 2.3. Statistical Analyses

The One-Sample Kolmogorov–Smirnov test with Lilliefors Significance Correction and Q–Q plots were used to study the data distribution. The concentrations of all biomarkers were not normally distributed (*p*-values < 0.01), except those of plasma betaine in the children and the mothers and plasma 5-MTHF in the fathers. All variables (including those with normal distribution) were log-transformed. The distribution was improved after log transformation and the log-transformed values were used for statistical tests that assume normal distribution of the data.

One-way analysis of variance (ANOVA) test was used to compare the log-transformed variables between more than two groups. The post hoc Tamhane T2 test was used to correct for multiple comparisons when ANOVA test showed an overall *p*-value ≤ 0.05 and the population variance was not homogenous (*p* < 0.05 from Levene statistics). Mann–Whitney test was used to compare continuous variables between two independent groups. Chi-square test was used to investigate differences in the distribution of categorical variables between two groups.

Logistic regression analyses were used to compute the odds ratio (OR) of the child to have a severe form of CHD when one of the parents had plasma choline < 10 µmol/L. Moreover, Generalized Linear Models (GLM) were used to study the association between having a severe form of CHD and the child or parents’ plasma concentrations of choline, betaine, the sum of choline and betaine or folate that were used as continuous exposure variables to investigate whether associations may exist over the whole concentration range of these biomarkers.

The GLM regression that tested child exposures (i.e., choline, betaine, the sum of choline and betaine or folate) was adjusted for the age of the child. When studying maternal exposures, we adjusted for child age, mother age and smoking habits in the mother (yes or no). Alternatively, when studying paternal exposures the GLM regression was adjusted for child age and father age. We decided not to adjust for additional self-reported variables, especially those related to the index pregnancy because of potential reporting bias due to the time distance. Each GLM regression model included one exposure variable (i.e., biomarker) or the same biomarker plus the above defined covariates. The EUROCAT system was used to classify the clinical severity of CHD and study the association with the biomarkers. The PAN classification was used in sensitivity analyses. Receiver Operating Characteristic (ROC) curve analyses were performed to illustrate the performance of parents’ plasma concentrations of choline in discriminating between clinically severe and mild CHD cases. The area under the curve (AUC) and its 95%CIs and the sensitivity and specificity of the optimal cutoff value are reported.

Additional sensitivity analysis included only families with non-syndromic CHD after excluding 14 cases with syndromic CHD [triosomy 21 (n = 8), with n = 1 case of each syndrome: Williams–Beuren syndrome (Chr7), Turner syndrome, microdillion 22q11, Ivemark’s syndrome, Heterotaxy syndrome, and CHARGE syndrome].

Multivariate linear regression analysis was used to explore determinants of urinary betaine concentrations in the child (outcome variable). We investigated whether child age, plasma folate, plasma choline, or severity of the CHD lesion were significant predictors of urinary betaine. Multicolinearity in linear regression analysis was studied by calculating the Variance Inflation Factor (VIF) that was below 1.3 for all variables in this model, thus indicating no colinearity.

We used version 31 of IBM^®^ SPSS^®^ Statistics package (SPSS Inc., Chicago, IL, USA). *p*-values ≤ 0.05 were considered statistically significant.

## 3. Results

### 3.1. Characteristics of the Study Population

Seventy-five children fulfilled the inclusion criteria and none of the exclusion criteria. Parents’ consent was available from 72 children. Among the patents, 69 mothers and 64 fathers agreed to fill the study questionnaire. Blood samples were available from 59 children, 57 mothers and 43 fathers. In total, 30 families contributed to the study with blood samples from children and both parents.

The mean (SD) age of the children was 3.1 (3.2) years (59.7% were boys). According to the EUROCAT classification, 45 children (62.5%) were diagnosed with severe CHD lesions. Based on the PAN study classification, 17 children (23.6%) had mild CHD, 22 (30.5%) had moderate CHD and 33 (45.8%) had severe CHD (Table 1). The mean (SD) maternal age was 32.1 (4.3) years at the birth of the index child and 35.0 (4.4) years at study recruitment. Seventeen mothers (24.6%) reported current smoking and only one woman reported drinking alcohol during pregnancy. The mean (SD) paternal age at recruitment was 37.8 (6.5) years. Additional characteristics of the parents are reported in Table S1.

Table 2 summarizes the distribution of specific CHD diagnoses and their categorization according to the EUROCAT and PAN classifications.

### 3.2. Concentrations of Choline, Betaine and Folate in Relation to CHD Severity

Plasma concentrations of choline and folate biomarkers in the children did not differ according to CHD severity (Table 3). In addition, plasma concentrations of choline, betaine, and folate did not differ between boys and girls. Urinary betaine concentrations were significantly higher in children with moderate and severe CHD compared with those with mild CHD, based on the PAN classifications (Table S2).

Maternal plasma concentrations of choline, betaine, and folate did not differ according to disease severity in the child, although choline concentrations tended to be lower in mothers of children with severe CHD (Table 3 and Table S2). Paternal plasma choline concentrations were significantly lower in fathers of children with severe CHD compared with fathers of children with mild CHD, according to EUROCAT and PAN classifications (Table 3 and Table S2). Plasma choline concentrations below 10 µmol/L in adults may indicate low choline intake [34,35]. A higher proportion of mothers (77% vs. 50%, *p* = 0.046 Chi-square test) and fathers (81% vs. 35%, *p* = 0.004 Chi-square test) had plasma choline concentrations below 10 µmol/L in the group with severe CHD compared to that with mild CHD (Table 3).

We further examined the associations between parental plasma biomarkers and CHD severity in the children (Table 4 and Table S3). Maternal plasma choline concentrations below 10 µmol/L were associated with a 3.7-fold higher risk of severe CHD in the child compared with concentrations ≥10 µmol/L [adjusted OR (95%CIs): 3.7 (1.1, 12.2) for severe CHD according to EUROCAT; 3.3 (1.0, 11.0) according to the PAN classification]. Similarly, low paternal plasma choline were associated with an increased risk of severe CHD in the child [adjusted OR (95%CIs): 7.4 (1.7, 31.5) for severe CHD according to EUROCAT (Table 4) and 4.0 (0.9, 17.4) according to the PAN classification] (Table S3).

The cutoff for plasma choline (i.e., 10 µmol/L) is not based on strong evidence; neither aims to draw conclusions on choline sufficiency. Therefore, we analyzed the association between CHD severity and plasma choline as a continuous variable. Parental plasma choline concentrations showed significant associations with CHD severity in the child, suggesting that the association is present across the range of plasma choline (Table 4). After excluding 14 cases with different syndromes, lower plasma choline concentrations were also observed in fathers of children with more severe CHD compared to those with mild CHD [median (IQR) = 6.0 (3.6) vs. 14.0 (8.3), *p* = 0.003 Mann–Whitney test] (Table S4). Maternal choline concentrations were slightly lower in the severe CHD group compared to the mild CHD group, but the difference was not significant.

Plasma concentrations of choline were available from 37 mother–father pairs. In 19 families, both parents had low plasma choline concentrations; among these, 16 children had severe CHD (EUROCAT classification). In contrast, among the 18 families in which only one parent had low choline concentrations or both parents had normal levels, only six children had severe CHD.

**Figure 1:**
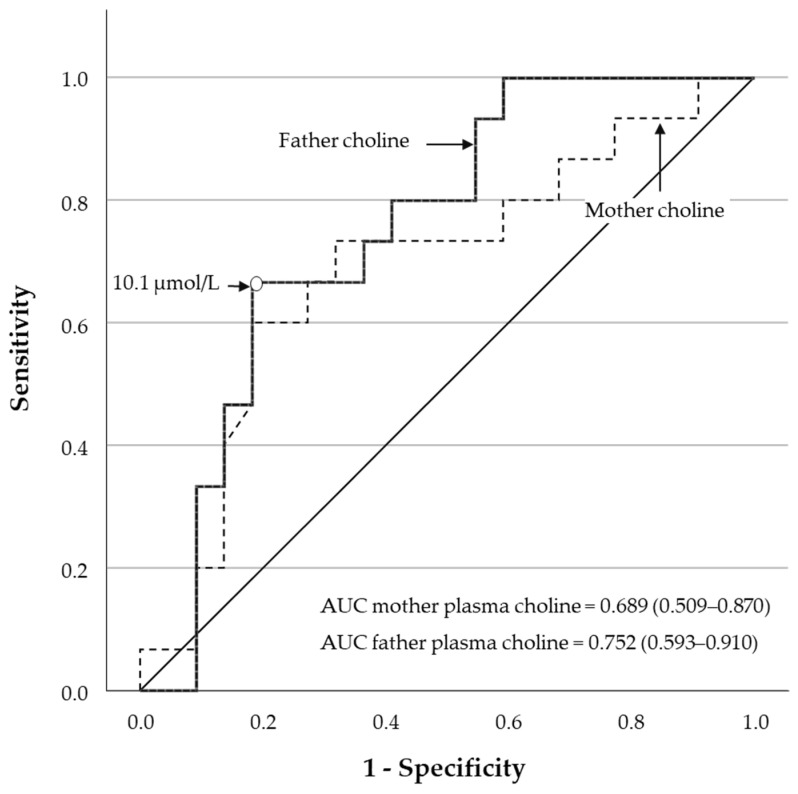
Receiver Operating Characteristics (ROC) curve analyses for using parents’ plasma concentrations of choline to discriminate between clinically severe and mild CHD cases according to EUROCAT classification. The area under the curve (AUC) and 95%CIs are slightly higher for paternal plasma choline. The optimal cut-off value for highest sensitivity and specificity in the present dataset was 10.1 µmol/L.

ROC curve analyses showed a slightly higher AUC for paternal plasma choline [0.752 (0.593–0.910)] compared to maternal plasma choline [0.689 (0.509–0.870)]. A paternal plasma choline cut-off value of 10.1 µmol/L provided 67% sensitivity and 82% specificity in distinguishing between severe and mild CHD according to EUROCAT classification. The same threshold value for maternal plasma choline would offer 60% sensitivity and 82% specificity (Figure 1).

No associations were observed between plasma betaine or folate concentrations (either in the children or in the parents) and CHD severity. Five mothers and two fathers reported adhering to a vegetarian diet and 17 mothers and six fathers were low meat eaters. No associations were found between the frequency of consuming meat, fish, and dairy products (per week) in the parents and plasma concentrations of choline or the severity of CHD in the child.

### 3.3. Interaction Between Choline and Folate Markers in the Children

Plasma choline and urinary metabolite concentrations in the children were analyzed according to tertiles of plasma folate. Higher concentrations of plasma folate were associated with higher concentrations of choline in plasma and betaine in urine (Table S5). In contrast, concentrations of plasma betaine were not associated with plasma folate (Table S4). In multivariable linear regression analyses, higher urinary betaine concentrations were predicted by CHD severity and higher concentrations of folate and choline. The child’s age was not a significant determinant of urinary betaine levels (Table S6).

## 4. Discussion

The present study examined biomarkers of choline and folate in blood samples from children with CHD and their parents in relation to CHD severity. We found that lower plasma choline concentrations in the parents, but not in the children were associated with severe CHD in the offspring. Notably, the likelihood of severe CHD appeared to be highest when both parents had plasma choline concentrations below 10 µmol/L, compared to families in which choline levels were normal in both parents or lowered in only one parent. The findings suggest additive effects of maternal and paternal risk factors and a dose–response relationship between low parental plasma choline concentrations and CHD severity.

The lack of associations between child plasma choline levels and CHD severity at age of 3 years may be explained by a generally high plasma choline in child age [36], excess choline oxidation to betaine and betaine excretion in urine. In contrast, the associations between lower choline concentrations in the parents and CHD severity in the child were detectable several years after conceiving the child, thus questioning whether these associations may reflect a familial pattern of disturbed choline metabolism [27,28]. In addition, dietary patterns in affected families may be stable over time and may increase the risk of severe CHD through interactions with genetic predispositions, but this has not been investigated in relation to CHD.

Choline is an essential nutrient involved in liver function, lipid metabolism, and homocysteine remethylation. The European Food Safety Authority (EFSA) recommends a daily choline intake of 400 mg for non-pregnant women and men and 480 mg for pregnant women [37]. The U.S. National Academy of Medicine recommends 550 mg/day for adults [37], with slightly lower requirements for young non-pregnant women due to estrogen-stimulated endogenous choline synthesis [38]. However, epidemiological studies indicate that dietary choline intake is on average 100–200 mg/day below these recommendations [39,40]. A recent observational study from China reported that higher choline intake during pregnancy was associated with a lower risk of CHD [41]. In that study, median total choline intake was low (190 mg/day in CHD cases and 247 mg/day in the controls). In addition, each 50 mg/day increase in choline intake was associated with 15% lower CHD risk [41]. Currently, most prenatal supplements do not contain choline, and there are no formal recommendations regarding paternal choline supplementation during preconception. Our findings, together with previous genetic and nutritional studies [27,28], support further investigation into whether optimizing parental choline status before and during pregnancy could reduce the risk of severe CHD. Moreover, whether increasing choline intake from foods or food supplements can influence different stages of cardiac development remains to be clarified.

Prevention of first occurrence and recurrent CHD and identification of risk factors in prospective parents are not well established. Wide-scale genetic testing is not cost effective. However, disorders in the BHMT pathway can be detected by measuring homocysteine after oral methionine load (e.g., 5.6 g to 7.5 g methionine for a 75 kg person), even when fasting plasma homocysteine is normal [42,43,44]. Post-methionine load hyperhomocysteinemia is a sensitive marker of insufficient choline intake [45,46,47] and it holds potential as a screening test that enables better targeting of C1 metabolism in high-risk couples. Future studies may explore whether increasing folate and choline intakes may lower the risk of severe CHD.

An additional novel finding of this study was the association between higher urinary betaine excretion and higher plasma folate concentrations in children with CHD. One possible explanation is a shift in the homocysteine remethylation pathway depending on folate availability. Under conditions of high folate status, increased activity of the folate-dependent remethylation pathway via methionine synthase may spare dietary choline and betaine, resulting in greater urinary excretion of betaine. This hypothesis requires further mechanistic investigation.

Although the present study cannot establish causality due to the time point of measuring circulating choline, experimental evidence supports a role for sufficient choline intake in normal cardiac development. Accordingly, choline deficiency in mice fed a choline-deficient diet for 6 weeks before mating [20] has been shown to cause heart defects including ventricular septal defects and impair normal cardiac development compared to animals on a standard diet [20]. In mouse models with impaired folate metabolism due to knockout of the methylenetetrahydrofolate reductase (MTHFR) gene, adequate maternal choline intake has been shown to partially rescue cardiac development [20]. Furthermore, choline derivatives and betaine have shown cardioprotective effects under conditions of prenatal stress or injury [48]. In a model of prenatal alcohol exposure, betaine supplementation (a product of choline oxidation) has been shown to contribute to normal development of great vessel diameters, interventricular septum thickness, and atrioventricular leaflet volumes [21], thus showing analogy to a possible role of choline in normal heart development. Collectively, these findings support a biologically plausible role for choline in embryonic heart development.

The present study has some limitations. First, plasma concentrations of choline were measured several years after birth and they may therefore not represent choline metabolism during pregnancy. Second, the classifications of CHD severity according to the EUROCAT or PAN study are designed to facilitate treatment, but they do not consider etiological factors or stages of fetal heart development. Third, the small sample size may cause incorrect inference due to Type 1 error (i.e., finding an association that does not exist). Finally, we do not have data on the dietary intake of choline or genetic polymorphisms [49] that may increase the demands of choline. However, on the one hand, the direction and magnitude of the association of maternal and paternal plasma choline and CHD severity are consistent. On the other hand, the dose–response relationship between disease severity and parents’ choline concentrations is unlikely to be due to chance, diet or lifestyles.

## 5. Conclusions

In conclusion, low parental plasma choline concentrations were associated with an increased risk of severe CHD in the offspring, with the highest risk observed when both parents had low choline levels. In contrast, the child’s own plasma choline concentrations were not related to lesion severity. Future studies integrating metabolic profiling with genetic analyses at early pregnancy may help identify modifiable risk factors and clarify the potential role of insufficient parental choline status in the development of severe CHD. Etiological studies aiming at the prevention of prevalent congenital anomalies should focus on maternal and paternal risk factors during pregnancy planning and early pregnancy.

## Figures and Tables

**Table 1:** Main characteristics of children with CHD and their parents.

Characteristics	All
Child age, years (n = 72)	3.1 (3.2)
Boys, n (%)	43 (59.7%)
Child height, cm (n = 66)	84 (27)
Child body weight, kg (n = 69)	12 (8)
Severity of CHD (EUROCAT classification), n (%)	
Mild	27 (37.5%)
Severe	45 (62.5%)
Severity of CHD (PAN study Classification) ^1^, n (%)	
Mild	17 (23.6%)
Moderate	22 (30.6%)
Severe	33 (45.8%)
CHD with any syndrome ^2^, n (%)	14 (19.4%)
CHD with Down syndrome	8 (11.1%)
Mother age at birth of the index child, years (n = 66)	32.1 (4.3)
Mother age at enrollment, years (n = 69)	35.0 (4.4)
Mother BMI at enrollment, kg/m^2^ (n = 65)	26.9 (6.8)
Mother currently smoker, n (%)	17 (24.6%)
Parity, n (%)	
1	26 (37.7%)
2	25 (36.2%)
≥3	18 (26.1%)
Father age at enrollment, years (n = 64)	37.8 (6.5)
Father BMI at enrollment, kg/m^2^ (n = 57)	27.4 (3.9)

Data are shown as mean (SD), unless otherwise specified. ^1^ PAN study classification as published before [1]. BMI, body mass index; CHD, congenital heart defects; EUROCAT, European network of population-based registries for the epidemiological surveillance of congenital anomalies. n, number of subjects. ^2^ Children with syndromes other than triosomy 21 were [1 case each: Williams–Beuren syndrome (Chr7), Turner syndrome, microdillion 22q11, Ivemark’s syndrome, Heterotaxy syndrome, and CHARGE syndrome].

**Table 2:** Diagnosis of CHD and clinical severity according to EUROCAT and the PAN Study.

CHD Type	N Cases	CHD Severity	
		EUROCAT Classification [30]	PAN Study Classification [1]
Atrial septal defect (ASD II), sinus venosus atrial septal defect (SVASD)	11	mild	mild
Ventricular septal defect (VSD)	11	mild	mild (when small or muscular VSD); moderate (for all other VSD)
Single ventricle	8	severe	severe
Complex CHD, not classifiable	6	severe	severe
Tetralogy of Fallot (TOF)	6	severe	severe
Atrioventricular septal defect (AVSD)	5	severe	moderate
Transposition of the great arteries (TGA)	5	severe	severe
Coarctation of the aorta (CoA)	6	severe	moderate
Aortic stenosis (AS)	3	severe	moderate
Anomalous pulmonary venous return (APVR)	2	severe	moderate (partial anomalous pulmonary venous return, PAPVC); severe (total anomalous pulmonary venous return, TAPVC)
Pulmonary atresia (PA)	2	severe	severe
Tricuspid atresia (TA)	2	severe	severe
Patent ductus arteriosus (PDA)	3	mild	mild
Truncus arteriosus communis (TAC)	1	severe	severe
Hypoplastic left heart syndrome (HLHS)	1	severe	severe

**Table 3:** Concentrations of choline and folate markers shown as mean (SD); median [IQR] in children with congenital heart defect (CHD) and their parents in total and according to EUROCAT classification of CHD severity.

	All	Mild CHD	Severe CHD	*p* ^1^
Child variables				
Age, weeks	3.1 (3.2); 2.1 [4.0]	2.7 (2.5); 1.7 [3.6]	3.3 (3.5); 3.4 [4.2]	0.972
Boys, n (%)	43 (59.7%)	13 (48.1%)	30 (66.7%)	0.142
*p*-choline, µmol/L (n = 56)	14.0 (10.0); 11.4 [8.9]	16.1 (12.8); 14.1 [9.4]	13.0 (8.4); 12.3 [8.5]	0.261
*p*-choline < 10 µmol/L, n/total	20/56	4/18	16/38	0.233 ^2^
*p*-betaine, µmol/L (n = 57)	52.7 (17.5); 51.8 [27.9]	53.4 (16.5); 54.9 [21.2]	52.4 (18.2); 50.3 [31.1]	0.520
Sum of *p*-choline and betaine, µmol/L (n = 56)	66.8 (22.4); 60.8 [31.0]	69.8 (24.6); 69.0 [34.8]	65.4 (21.4); 59.6 [29.9]	0.405
Urine choline, mmol/mol creatinine (n = 57)	1.54 (2.44); 0.7 [1.2]	2.0 (3.4); 0.8 [1.8]	1.2 (1.6); 0.7 [1.0]	0.589
Urine betaine, mmol/mol creatinine (n = 58)	68.1 (100.1); 9.0 [104.2]	72.0 (123.9); 7.3 [110.9]	65.7 (84.3); 30.9 [105.9]	0.423
*p*-total folate, nmol/L (n = 59)	12.6 (11.3); 10.2 [12.5]	12.5 (13.5); 6.7 [12.1]	12.6 (10.1); 11.1 [12.6]	0.442
*p*-5-MTHF, nmol/L (n = 59)	10.8 (9.4); 7.2 [11.4]	11.1 (11.7); 5.7 [11.3]	10.6 (8.1); 8.8 [11.8]	0.608
*p*-non-methyl-THF, nmol/L (n = 59)	1.8 (5.6); 0.5 [1.0]	1.4 (2.6); 0.8 [1.3]	2.0 (6.7); 0.4 [0.7]	0.586
Mother plasma biomarkers				
Choline, µmol/L (n = 57)	9.5 (5.1); 7.7 [5.5]	10.5 (4.7); 9.9 [7.9]	8.8 (5.2); 7.4 [3.9]	0.096
Choline < 10 µmol/L, n/total	38/57	11/22	27/35	0.046 ^2^
Betaine, µmol/L (n = 57)	36.0 (12.6); 34.8 [17.0]	36.4 (13.2); 37.4 [15.4]	35.8 (12.4); 33.3 [15.6]	0.682
Sum of choline and betaine, µmol/L (n = 57)	45.5 (14.8); 46.3 [16.6]	47.0 (13.9); 47.5 [15.1]	44.6 (15.5); 41.9 [19.4]	0.517
Total folate, nmol/L (n = 65)	7.4 (7.2); 4.9 [5.5]	5.1 (3.9); 3.7 [3.1]	8.8 (8.3); 5.3 [10.8]	0.126
5-MTHF, nmol/L (n = 65)	6.2 (5.8); 4.2 [4.2]	4.3 (3.8); 3.1 [3.0]	7.3 (6.5); 4.9 [8.8]	0.087
Non-methyl-THF, nmol/L (n = 65)	1.2 (3.2); 0.3 [0.7]	0.7 (1.3); 0.2 [0.9]	1.6 (3.9); 0.3 [0.7]	0.780
Father plasma biomarkers				
Choline, µmol/L (n = 43)	10.3 (5.4); 8.1 [9.2]	12.2 (4.5); 10.7 [8.5]	9.1 (5.7); 7.1 [4.0]	0.008
Choline < 10 µmol/L, n/total	27/43	6/17	21/26	0.004 ^2^
Betaine, µmol/L (n = 44)	41.5 (10.8); 38.1 [10.2]	40.5 (9.4); 37.1 [9.3]	42.2 (11.8); 39.7 [12.4]	0.772
Sum of choline and betaine, µmol/L (n = 43)	51.9 (12.7); 48.7 [16.3]	52.8 (11.9); 49.8 [16.7]	51.3 (13.4); 48.6 [15.3]	0.494
Total folate, nmol/L (n = 50)	3.8 (3.0); 3.4 [2.0]	2.9 (1.4); 2.5 [2.2]	4.4 (3.5); 3.6 [2.4]	0.050
5-MTHF, nmol/L (n = 50)	3.1 (1.7); 2.8 [2.0]	2.5 (1.2); 2.4 [2.0]	3.5 (1.9); 3.4 [2.1]	0.069
Non-methyl-THF, nmol/L (n = 50)	0.7 (2.6); 0.1 [0.4]	0.3 (0.3); 0.3 [0.5]	0.9 (3.3); 0.1 [0.3]	0.161

Results are shown as mean (standard deviation, SD); median [interquartile, IQR] unless otherwise specified. ^1^ *p*-values from the Mann–Whitney U Test. ^2^ Chi-square test was used to compare categorical variables. CHD, congenital heart defect EUROCAT, European network of population-based registries for the epidemiological surveillance of congenital anomalies; n, number of observations; 5-MTHF, 5-methyltetrahydrofolate; *p*-choline, plasma choline.

**Table 4:** Associations of child, maternal and paternal biomarker concentrations with severity of CHD according to EUROCAT classification.

Logistic Regression (Exposure *p*-Choline < 10 µmol/L)	Number Mild/Severe CHD	Crude OR (95%CIs)	Adjusted OR (95%CIs) ^1,2^
Mother *p*-choline ≥ 10 µmol/L (n = 19 of 57 mothers)	11/8	OR = 1	
Mother *p*-choline < 10 µmol/L (n = 38 of 57 mothers)	11/27	3.7 (1.2, 11.9)	3.7 (1.1, 12.2)
Father *p*-choline ≥ 10 µmol/L (n = 16 of 43 fathers)	11/5	OR = 1	
Father *p*-choline < 10 µmol/L (n = 27 of 43 fathers)	6/21	7.7 (1.9, 31.0)	7.4 (1.7, 31.5)
Mother and/or father’s *p*-choline ≥ 10 µmol/L, n = 18	12/6	OR = 1	
Both mother and father’s *p*-choline < 10 µmol/L, n = 19	3/16	10.7 (2.2, 51.5)	11.1 (2.1, 59.3)
Generalized Linear Models (exposure: log-transformed biomarkers)		β-coefficient (95%CIs)	Adjusted β-coefficient (95%CIs) ^1,2^
Mother *p*-choline		−0.53 (−1.12, 0.07)	−0.61 (−1.20, −0.02) ^3^
Mother *p*-betaine		0.04 (−0.67, 0.75)	−0.06 (−0.77, 0.65)
Mother *p*-folate		0.23 (−0.04, 0.62)	0.32 (−0.02, 0.65)
Father *p*-choline		−0.85 (−0.49, −0.20)	−0.87 (−1.58, −0.16) ^3^
Father *p*-betaine		0.31 (−1.13, 1.76)	0.21 (−1.23, 1.65)
Father *p*-folate		0.54 (0.07, 1.01)	0.50 (0.01, 0.99)
Child *p*-choline		−0.30 (−0.77, 0.17)	−0.317 (−0.80, 0.17)
Child *p*-betaine		−0.06 (−0.85, 0.73)	−0.04 (−0.88, 0.80)
Child *p*-folate		0.11 (−0.20, 0.41)	0.16 (−0.17, 0.49)

^1^ The regression models of maternal exposures are adjusted for age of the mother, age of the child and maternal smoking (yes or no). ^2^ The regression models of paternal exposures are adjusted for age of the father and age of the child. ^3^ The GLM regression shows an inverse association between choline concentrations and CHD severity. Thus, lower choline concentrations were associated with severe CHD (CHD forms were coded as 1 (for mild CHD) and 2 (for severe CHD) according to EUROCAT classification). OR and (95%CIs) were computed by running logistic regression analyses with the severity of the CHD entered in the model as an outcome variable (1 = mild; 2 = severe). The exposure variable (plasma choline in the mother or the father < 10 µmol/L vs. ≥10 µmol/L) and the model-specific covariates were entered as independent variables. The beta coefficient (β) and 95%CIs were computed from a Generalized Linear Model (GLM) using the severity of the CHD as an outcome variable and one exposure variable (e.g., log-transformed plasma choline in the mother or the father) or the same exposure factor plus the covariates as predictor variables. Results in bold are statistically significant. CHD, congenital heart defect; EUROCAT, European network of population-based registries for the epidemiological surveillance of congenital anomalies; n, number of subjects; *p*-choline (betaine or folate), plasma choline (betaine or folate).

## Data Availability

Data can be provided in aggregate form to scientists upon reasonable request to the corresponding author.

## Supplementary Materials

The following supporting information can be downloaded at: https://www.mdpi.com/article/10.3390/nu18091455/s1, Table S1: Description of parents’ health and lifestyle factors according to severity of CHD in the child. Table S2: Plasma concentrations of choline and folate markers in children with congenital heart defect (CHD) and their parents. The data are shown for the whole group and by severity of CHD according to the PAN study classification. Table S3: Associations of maternal and paternal biomarker concentrations with severity of CHD in the child classified according to the PAN study. Table S4: Concentrations of choline and folate in families with mild or severe CHD (EUROCAT classification) in the subgroup of participants without syndromic CHD. Table S5: Plasma and urinary betaine and choline concentrations in children with CHD according to tertiles of plasma folate in the children. Table S6: Multivariate linear regression analyses to predict urinary concentrations of betaine in children with congenital heart defect (as an outcome variable).

## Abbreviations

The following abbreviations are used in this manuscript:

BHMT	betaine-homocysteine methyltransferase
CHD	congenital heart defect
EFSA	European Food Safety Authority
EUROCAT	European network of population-based registries for the epidemiological surveillance of congenital anomalies
5-MTHF	5-methyltetrahydrofolate
MTHFR	methylenetetrahydrofolate reductase
PAN	Praevalenz angeborener Herzfehler bei Neugeborenen
