# Supplementary material for: Lowered Maternal and Paternal Plasma Concentrations of Choline Are Associated with the Severity of Congenital Heart Defects in the Offspring
Source: Nutrients. 2026 May 1;18(9):1455. doi: 10.3390/nu18091455 (PMC13165152; doi:10.3390/nu18091455)
Supplement: Supplementary file 1 [file nutrients-18-01455-s001.zip › nutrients-4186413-supplementary.pdf]

Lowered maternal and paternal plasma concentrations of choline are associated with the severity of congenital heart defects in the offspring

Rima Obeid[1*], Annabelle Wagner[2,3], Celina Löhfeln[1], Jürgen Geisel[1], Hashim Abdul-Khaliq[3]

[1] Saarland University Hospital, Department of Clinical Chemistry and Laboratory Medicine, Kirrberg Street, Building 57, D-66421, Homburg/Saar, Germany. Telephone: 0049-6841-1630711, Email: rima.obeid@uks.eu; Celina.Loehfelm@uks.eu; Juergen.geisel@uks.eu

[2] Saarland University Hospital, Department of Pediatric Hematology and Oncology, Kirrberger Straße, Building 9, D-66421, Homburg/Saar, Germany. Email: Annabelle.Wagner@uks.eu

[3] Saarland University and University Hospital, Department of Pediatric Cardiology, Building 9, D-66424, Homburg/Saar, Germany. Email: Hashim.Abdul-Khaliq@uks.eu

* Correspondence: Prof. Dr. Rima Obeid

Supplementary tables

Table S1. Description of parents' health and lifestyle factors according to severity of CHD in the child.

	EUROCAT classification	
	**Mild (n = 27)**	**Severe (n = 45)**
**Maternal factors**		
Use of multivitamin supplements containing folate any time during the index pregnancy, n(%)	21 (87.5%)	25 (69.4%)
Current use of multivitamin supplements with folate, n(%)	5 (20.0%)	11 (26.2%)
Vegan diet, n(%)	1 (3.8%)	4 (9.3%)
Present alcohol consume (3-7 drinks/w), n(%)	2 (8.7%)	1 (2.6%)
Alcohol consume during pregnancy, n(%)	0	1 (2.4%)
Smoked before pregnancy, n(%)	8 (30.7%)	7 (17.1%)
Mother born outside Germany, n(%)	4 (14.8%)	9 (20.0%)
Worked during pregnancy (part or full time), n(%)	13 (52.0%)	22 (52.4%)
**Paternal factors**		
Father born outside Germany, n(%)	5 (20.8%)	13 (39.4%)
Present alcohol consume, n(%)		
3-7 drinks per week	3 (11.1%)	6 (13.3%)
>7 drinks per week	1 (3.7%)	2 (4.4%)

Table S2. Plasma concentrations of choline and folate markers in children with congenital heart defects (CHD) and their parents. The data are shown for the whole group and by severity of CHD according to the **PAN study classification** [1].

	All	PAN classification			P (ANOVA)[1]
		Mild	Moderate	Severe	
**Child**					
Age, weeks	3.1 (3.2); 2.1 [4.0]	3.9 (2.6); 3.4 [4.1]	3.0 (3.9); 1.3 [3.6]	2.7 (2.9); 1.0 (3.9);	0.431
Plasma choline, µmol/L (n = 56)	14.0 (10.0); 11.4 [8.9]	11.4 (4.4); 10.8 [3.8]	19.6 (15.7); 13.8 [18.2]	12.2 (6.6); 10.5 [7.2]	0.137
Plasma choline < 10 µmol/L, n/total	20/56	4/12	4/15	12/29	0.616[2]
Plasma betaine, µmol/L (n = 57)	52.7 (17.5); 51.8 [27.9]	48.9 (16.7); 51.8 [26.5]	51.1 (16.1); 47.1 [27.3]	55.2 (18.7); 53.7 [30.2]	0.528
Urine choline, mmol/mol creatinine (n = 57)	1.54 (2.44); 0.7 [1.2]	0.8 (0.6); 0.6 [0.8]	2.3 (3.6); 1.2 [2.1]	1.4 (1.8); 0.6 [1.1]	0.298
Urine betaine, mmol/mol creatinine (n = 58)	68.1 (100.1); 9.0 [104.2]	20.9 (53.7); 4.0 [6.0]	112.3 (134.1); 84.9 [229.0][3]	59.4 (76.2); 31.3 [93.1][3]	0.024
Plasma total folate (sum of folate forms), nmol/L (n = 59)	12.6 (11.3); 10.2 [12.5]	11.4 (11.9); 7.2 [10.3]	12.1 (11.3); 7.4 [12.6]	13.5 (11.3); 11.2 [20.0]	0.840
Plasma 5-MTHF, nmol/L (n = 59)	10.8 (9.4); 7.2 [11.4]	11.2 (10.6); 5.9 [9.9]	9.9 (9.3); 7.2 [12.3]	10.8 (8.8); 8.5 [12.6]	0.839
Plasma non-methyl-THF, nmol/L (n = 59)	1.8 (5.6); 0.5 [1.0]	1.5 (3.0); 0.7 [1.1]	0.9 (1.1); 0.5 [1.0]	2.6 (8.0); 0.4 [0.7]	0.580
**Mother markers**					
Plasma choline, µmol/L (n = 57)	9.5 (5.1); 7.7 [5.5]	10.2 (5.0); 8.7 [8.0]	9.9 (4.1); 9.3 [5.1]	8.7 (5.7); 7.3 [2.8]	0.257
Plasma choline < 10 µmol/L, n/total	38/57	8/14	10/18	20/25	0.168[2]
Plasma betaine, µmol/L (n = 57)	36.0 (12.6); 34.8 [17.0]	38.2 (13.4); 38.3 [15.3]	36.9 (14.0); 36.1 [24.5]	34.2 (11.4); 33.3 [13.8]	0.852
Plasma total folate (sum of folate forms), nmol/L (n = 65)	7.4 (7.2); 4.9 [5.5]	4.7 (3.3); 3.7 [2.6]	8.3 (9.0); 5.1 [3.9]	8.4 (7.2); 6.0 [9.4]	0.229
Plasma 5-MTHF, nmol/L (n = 65)	6.2 (5.8); 4.2 [4.2]	4.2 (3.1); 2.9 [3.3]	6.6 (7.3); 4.9 [4.2]	7.0 (5.8); 4.7 [8.9]	0.332
Plasma non-methyl-THF, nmol/L (n = 65)	1.2 (3.2); 0.3 [0.7]	0.5 (0.6); 0.3 [0.7]	1.7 (3.9); 0.3 [1.2]	1.4 (3.5); 0.3 [0.8]	0.516
**Father markers**					
Plasma choline, µmol/L (n = 43)	10.3 (5.4); 8.1 [9.2]	12.7 (4.6); 15.0 [9.5]	10.0 (4.7); 8.1 [4.6]	9.2 (6.3); 6.2 [4.1]	0.108
Plasma choline < 10 µmol/L, n/total	27/43	3/10	11/17	13/16	0.031[2]
Plasma betaine, µmol/L (n = 44)	41.5 (10.8); 38.1 [10.2]	43.1 (11.3); 39.3 [18.5]	40.6 (12.0); 36.9 [9.9]	41.6 (9.9); 39.7 [11.8]	0.806
Plasma total folate (sum of folate forms), nmol/L (n = 50)	3.8 (3.0); 3.4 [2.0]	2.7 (1.1); 2.5 [2.0]	5.1 (4.3); 3.9 [2.9]	3.4 (1.7); 3.3 [2.1]	0.189
Plasma 5-MTHF, nmol/L (n = 50)	3.1 (1.7); 2.8 [2.0]	2.5 (1.0); 2.4 [2.0]	3.4 (2.1); 3.2 [3.2]	3.2 (1.7); 3.0 [1.9]	0.744

Plasma non-methyl-THF, nmol/L (n = 50)	0.7 (2.6); 0.1 [0.4]	0.2 (0.3); 0.1 [0.3]	1.6 (4.2); 0.5 [0.9]	0.2 (0.3); 0.1 [0.2]	0.173

Results are shown as mean (SD); median [IQR] unless otherwise specified.

[1] P value for between-group comparison of continuous variables is according to ANOVA test applied using the log-transformed values.

[2] Chi-square test was used to compare categorical variables between the groups.

[3] both moderate and severe CHD forms are significantly different from the mild CHD forms using post-hoc Tamhane test.

Table S3. Associations of child, maternal and paternal biomarker with severity of CHD classified according to the PAN study [1].

	Classification of CHD severity according to the PAN study		
**Logistic regression analyses**	**N mild or moderate/n severe CHD**	**Crude OR (95%CI)**	**Adjusted OR (95%CI) [1,2]**
Mother plasma choline concentrations ≥ 10 µmol/L (n = 19 of 57 mothers)	14/5	OR = 1	
Mother plasma choline concentrations < 10 µmol/L (n = 38 of 57 mothers)	18/20	3.3 (1.0, 11.0)	3.5 (1.0, 12.0)
Father plasma choline concentrations ≥ 10 µmol/L (n = 16 of 43 fathers)	13/3	OR = 1	
Father plasma choline concentrations < 10 µmol/L (n = 27 of 43 fathers)	14/13	4.0 (0.9, 17.4)	5.6 (1.1, 27.1)
Mother and/or father's plasma choline ≥ 10 µmol/L , n = 18	16/2	OR = 1	
Both mother and father's plasma choline < 10 µmol/L, n = 19	8/11	11.0 (2.0, 62.0)	22.9 (2.8, 190.0)
**Exposures as continuous variables (log-transformed) in GLM analyses**		**Beta coefficient (95%CI)**	**Adjusted beta coefficient[1,2] (95%CI)**
Mother plasma choline		-1.06 (-2.28, 0.15)	-1.23 (-2.45, 0.003)[3]
Mother plasma betaine		-0.42 (-1.87, 1.02)	-0.39 (-1.86, 1.08)
Mother plasma folate		0.44 (-0.25, 1.13)	0.38 (-0.33, 1.10)
Father plasma choline		-1.18 (-2.50, 0.15)	-1.69 (-3.11, -0.27)[3]
Father plasma betaine		0.18 (-2.71, 3.08)	0.43 (-2.46, 3.33)
Father plasma folate		-0.27 (-1.27, 0.74)	-0.14 (-1.19, 0.90)
Child plasma choline		-0.68 (-1.68, 0.32)	-0.93 (-1.93, 0.07)
Child plasma betaine		0.88 (-0.79, 2.55)	0.56 (-1.19, 2.30)
Child plasma folate		0.20 (-0.45, 0.85)	0.08 (-0.60, 0.77)

The beta coefficient and 95% confidence intervals were computed from a Generalized Linear Model (GLM) using the severity of the CHD as an outcome variable. The exposure variable (lowered plasma choline in the mother or the father) alone or with the other co-variates entered as predictor variables.

[1] regression models including maternal exposures are adjusted for age of the mother, age of the child and maternal smoking (yes or no).

[2] regression models including paternal exposures are adjusted for age of the father and age of the child.

[3] The linear regressions show an inverse association, thus lower plasma choline concentrations were associated with sever CHD forms.

OR and the 95% CIs were computed by logistic regression analyses where the severity of the CHD (1 = mild or moderate; 2 = severe) was entered as an outcome variable. The exposure variable (plasma choline in the mother or the father < vs. ≥ 10 µmol/L) and the covariates were entered as independent variables.

The beta coefficient and 95%CIs were computed from a Generalized Linear Model (GLM) using the severity of the CHD as an outcome variable. The exposure variable (e.g., log-transformed plasma choline in the mother or the father) alone or with the covariates entered as predictor variables.

Table S4. Concentrations of choline and folate in families with mild or severe CHD (EUROCAT classification) in the subgroup of participants without syndromic CHD.

Variables	Mild CHD Percentiles			Severe CHD Percentiles			P (Mann-Whitney)
	50	25	75	50	25	75	
Mother plasma choline, µmol/L	9.4	6.5	14.2	7.3	5.4	10.5	0.188
Mother plasma betaine, µmol/L	37.6	30.5	45.1	32.5	24.5	42.6	0.378
Mother total folate, nmol/L	3.8	2.9	5.8	5.8	3.3	13.6	0.088
**Father plasma choline, µmol/L**	**14.8**	**8.5**	**16.8**	**6.0**	**5.6**	**9.1**	**0.003**
Father plasma betaine, µmol/L	40.9	34.2	47.8	37.9	33.1	43.4	0.657
Father total folate, nmol/L	2.5	1.4	4.0	3.6	2.5	4.5	0.056
Child urine betaine, mmol/mol creatinine	5.3	3.0	129.1	45.3	4.6	110.6	0.502
Child urine choline, mmol/mol creatinine	.7	.4	2.2	.6	.4	1.4	0.356
Child plasma choline, µmol/L	12.0	9.7	17.8	11.1	7.4	18.6	0.482
Child plasma betaine, µmol/L	52.7	42.7	63.8	51.5	34.7	68.4	0.877
Child total folate, nmol/L	8.5	3.8	15.9	11.2	4.2	23.4	0.501

Table S5. Concentrations of betaine, choline and folate in children with CHD according to tertiles of plasma folate in the children.

	Tertile of plasma total folate in the child			
	Lowest tertile (n = 19)	Medium tertile (n =20)	Highest tertile (n =20)	p
Plasma folate, nmol/L median (min-max)	3.3 (1.2-4.9)	10.0 (4.9-12.6)	23.6 (13.5-48.4)	-
Plasma choline, µmol/L	8.5 [5.1]	10.1 [6.9]	14.5 [13.1]	0.033
Plasma betaine, µmol/L	42.7 [19.7]	53.2 [19.9]	53.2 [30.9]	0.235
Urine choline, mmol/mol creatinine	0.34 [0.37]	0.79 [1.00]	1.64 [2.30]	0.002
Urine betaine, mmol/mol creatinine	4.6 [5.8]	8.3 [58.8]	99.6 [110.0]	<0.001
Sum of plasma choline and betaine, µmol/L	50.7 [21.1]	66.6 [33.9]	73.3 [31.7]	0.063
Sum of urine choline and betaine, mmol/mol creatinine	4.8 [5.7]	8.9 [59.7]	98.8 [126.6]	<0.001

Data are median and [Interquartile range, IQR] unless otherwise specified.
p values are according to ANOVA test applied using the log transformed data.

Table S6. Multivariate linear regression analyses to predict log- urine concentrations of betaine in children with congenital heart defects (as an outcome variable)

Predictors of urine concentrations of betaine in children with CHD	Standardized beta coefficient and 95%CI	p
Age of the child	-0.16 (-0.002, 0.00)	0.221
Plasma choline	0.37 (0.30, 1.80)	0.007
Plasma folate	0.33 (0.15, 1.26)	0.014
Severity of CHD according to the PAN study[1]	0.26 (0.01, 0.47)	0.046

Continuous variables were entered in the regression model as log-transformed values.

[1] Severity of CHD is coded as 1,2,3 where 3 codes for the most severe forms.

Adjusted R^2 = 0.50. Multicolinearity in linear regression analysis was studied by calculating the Variance Inflation Factor (VIF) that was below 1.3 for all variables in this model, thus indicating no colinearity.
